# Tixagevimab and Cilgavimab (Evusheld) as Pre-exposure Prophylaxis for COVID-19 in Patients With Inflammatory Bowel Disease: A Propensity Matched Cohort Study

**DOI:** 10.1093/crocol/otad047

**Published:** 2023-09-06

**Authors:** Aakash Desai, Jana G Hashash, Gursimran S Kochhar, Francis A Farraye

**Affiliations:** Division of Gastroenterology and Hepatology, MetroHealth Medical Center, Case Western Reserve University, Cleveland, OH, USA; Division of Gastroenterology and Hepatology, Mayo Clinic, Jacksonville, FL, USA; Division of Gastroenterology & Hepatology, Allegheny Health Network, Pittsburgh, PA, USA; Division of Gastroenterology and Hepatology, Mayo Clinic, Jacksonville, FL, USA

**Keywords:** inflammatory bowel disease, COVID-19, tixagevimab, cilgavimab

## Abstract

**Background:**

Tixagevimab and cilgavimab (Evusheld) are 2 fully human monoclonal antibodies that received emergency-use authorization on December 21, 2021, for pre-exposure prophylaxis of coronavirus disease 2019 (COVID-19) in patients who are moderate–severely immunocompromised. The real-world efficacy of Evusheld in patients with inflammatory bowel disease (IBD) is not known.

**Methods:**

We conducted a retrospective cohort study using TriNetX, a multi-institutional database in patients with IBD who received Evusheld compared to patients with IBD who did not receive Evusheld (12.1.2021–10.28.2022). The primary outcome was to assess the risk of COVID-19 within 6 months. One-to-one propensity score matching (PSM) was performed for demographic parameters, comorbid conditions, IBD medications, and history of COVID-19. Risk was expressed as adjusted odds ratio (aOR) with 95% confidence interval (CI).

**Results:**

Four hundred and eight patients (0.19%) with IBD received Evusheld (mean age 58.6 ± 15.4 years old, female 47.7%) during the study period. After PSM, there was no difference in the risk (aOR 0.88, 95% CI, 0.33–2.35) of COVID-19 in the Evusheld cohort compared to the IBD control cohort. No patients required ICU care or intubation/respiratory support or were deceased in the Evusheld cohort.

**Conclusions:**

Our study did not show that Evusheld decreases the risk of COVID-19 in patients with IBD. Prevention of moderate–severe COVID-19 in these patients should focus on vaccination strategies and early COVID-19 therapies.

## Introduction

Tixagevimab and cilgavimab are 2 fully human monoclonal antibodies that simultaneously bind to distinct nonoverlapping epitopes of severe acute respiratory syndrome coronavirus-2 (SARS-CoV-2) spike-protein receptor-binding domain to neutralize the virus.^1^ The PROVENT randomized control trial concluded that the use of tixagevimab and cilgavimab in patients who were at an increased risk for inadequate response to coronavirus disease 2019 (COVID-19) vaccines had a lower risk of COVID-19.^2^ Although the efficacy of these monoclonal antibodies could not be estimated in the immunocompromised subgroup due to small sample size, the U.S. Food and Drug Administration (FDA) issued an emergency-use authorization for tixagevimab plus cilgavimab (Evusheld-AstraZeneca) on December 21, 2021, for pre-exposure prophylaxis (PrEP) of COVID-19 in patients who are moderate–severely immunocompromised.^3^ Two retrospective cohort studies have shown that tixagevimab and cilgavimab provide protection against COVID-19 in patients who are immunocompromised.^4,5^ The real-world efficacy of tixagevimab and cilgavimab in patients with inflammatory bowel disease (IBD) is not known. The primary aim of this brief report was to assess the efficacy of tixagevimab and cilgavimab in preventing COVID-19 in patients with IBD.

## Methods

### Database

A retrospective cohort study using the multi-institutional research network TriNetX (Cambridge, MA) was performed. TriNetX is a global federated research network that provides real-time access to de-identified electronic health records (EHRs) of more than 85 million patients within 56 healthcare organizations (HCOs) in the United States. The de-identification process is determined and done at a network level and attested through a formal determination by a qualified expert as defined in the HIPAA Privacy Rule. TriNetX obfuscates patient counts <11 to ensure patient anonymity. Informed consent was not required for this study as TriNetX is a de-identified database. Clinical variables are derived directly from EHRs of included HCOs as well as retrieved through a built-in natural language processing system that extracts variables from clinical documents. Robust quality assurance is achieved at the time of extraction from EHRs before inclusion in the database, in a systemic and standardized format. The interface only provides aggregate counts and statistical summaries to protect patient health information.

### Study Participants and Cohorts

A real-time search and analysis of the U.S. Collaborative Network in the TriNetX platform was conducted from January 1, 2022, through October 28, 2022. Patients with IBD were identified using the International Classification of Disease, Tenth Revision, Clinical Modification (ICD-10-CM) code for “Ulcerative colitis” (K51) or “Crohn’s disease” (K50) plus RxNorm codes for at least one IBD-related medication mesalamine, sulfasalazine, balsalazide, olsalazine, infliximab, adalimumab, certolizumab pegol, golimumab, vedolizumab, ustekinumab, tofacitinib, azathioprine, mercaptopurine, methotrexate, oral prednisone, or budesonide. Complex case definitions for identification of IBD cohort which include ≥1 ICD-10-CM code plus a relevant IBD-related prescription from administrative and claims databases has been shown to have ≥80% positive predictive value and ≥85% specificity.^6^ The Evusheld cohort included adult patients ≥18 years old with IBD who had Current Procedural Terminology (CPT) codes for injection of tixagevimab and cilgavimab. TriNetX allows defining index events and excluding patients with outcomes prior to the index event. This functionality was used to ensure the administration of Evusheld occurred after the diagnosis of IBD. Evusheld was approved during the Omicron variant period. The IBD control cohort included patients with ICD-10-CM codes for IBD between December 1, 2021, and March 31, 2022, who did not receive Evusheld. Only patients within this timeframe were included to ensure that the index event occurred during a period where the Omicron variant of SARS-CoV-2 was becoming the more prevalent variant. All patients had a minimum 6-month follow-up. Patients who received a COVID-19 vaccine during the follow-up period were excluded from the study.

### Study Outcomes

The primary outcome of the study was to assess the risk of COVID-19 between the Evusheld cohort and IBD control cohort within 6 months. TriNetX captures data on COVID-19 patients, their pre-existing risk factors, treatments, and outcomes. Patients with IBD who had COVID-19 were identified with COVID-19-specific laboratory Logical Observation Identifiers Names and Codes, positive SARS coronavirus-2 and related RNA, or ICD-10-CM code for COVID-19 (U07.1 or J12.92). This methodology has been used to identify patients with COVID-19 in prior published studies.^7^

The secondary outcomes were as follows:

- To assess the risk of any-cause hospitalization, intubation/respiratory support, intensive care unit (ICU) care, and all-cause mortality within 30 days of COVID-19 diagnosis or positive SARS-CoV-2 test. Status of hospitalization was based on the CPT code “Hospital Inpatient Services” (1013659). Status of death was based on the vital status code “Deceased” that TriNetX regularly imports from the Social Security Death index. Intubation or respiratory support was identified based on CPT code 31500 “Intubation, endotracheal, emergency procedure,” code 5A1945Z “Respiratory Ventilation, 24-96 hours Consecutive hours” or code 5A1955Z “Respiratory Ventilation, Greater than 96 Consecutive Hours.” ICU care was identified using CPT code 1013729 “Critical Care Services.”
- To assess the risk of COVID-19 in the Evusheld cohort compared to a non-IBD control cohort that received Evusheld. The non-IBD control cohort included patients without IBD, rheumatoid arthritis, psoriasis, systemic lupus erythematous, ankylosing spondylitis, or multiple sclerosis who had CPT codes for Evusheld.

### Statistical Analysis

All statistical analyses were conducted using the TriNetX software using the browser-based real-time analytics feature, TriNetx Live (TriNetX LLC, Cambridge, MA). Baseline characteristics of cohorts were described using means, standard deviations, and proportions. One-to-one (1:1) propensity score matching (PSM) was performed for age, gender, race, ethnicity, obesity, diabetes mellitus, nicotine dependence, chronic lower respiratory disease, ischemic heart disease, heart failure, chronic kidney disease, cerebrovascular accident, malignancy, IBD type, and disease location. Propensity score matching was also performed for steroid use, IBD medications, history of COVID-19, and ≥2 doses of COVID-19 vaccine within 1 year. TriNetX does not allow for 1:2 or 1:3 PSM. TriNetX platform utilizes input matrices of the user-identified covariates to conduct logistic regression analysis to obtain propensity scores for all individual subjects. The propensity scores generated are used to match patients using greedy nearest-neighbor algorithms with a caliper width of 0.1 pooled standard deviations. TriNetX randomizes the order of rows to eliminate bias resulting from nearest-neighbor algorithms. *P* values after PSM indicate the success of matching a covariate between the 2 cohorts. A complete list of covariates with corresponding *P* values before and after PSM can be found in Table S1. After PSM, risk of each outcome was expressed as adjusted odds ratio (aOR) with 95% confidence interval (CI). Two-sided *P* values <.05 were considered statistically significant.

## Results

### Characteristics of Study Population

Out of 211 563 patients with IBD, 408 patients (0.19%) received tixagevimab–cilgavimab (mean age 58.6 ± 15.4 years old, female 47.7%) during the study period. Two hundred and seventeen (53.1%) patients had Crohn’s disease. Eighty-nine patients (21.8%) had a history of COVID-19, 1 year prior. Only 22 patients (5.3%) received a COVID-19 vaccine within 6 months prior to tixagevimab–cilgavimab administration. There were 66 375 patients in the IBD control cohort (mean age 48.9 ± 18.5, female 54.9). 37 864 (57%) patients had Crohn’s disease. Table 1 shows the comparison between demographics, comorbid diseases, IBD type, and medications between the Evusheld and IBD control cohorts.

**Table 1.** Comparison of demographic parameters, comorbid diseases, IBD types, and IBD medications between patients in Evusheld cohort and IBD control cohort before and after propensity score matching.

Demographics	Before propensity score matching			After propensity score matching	Control cohort (*n* = 391)	*P* value
	Evusheld cohort (*n* = 408)	Control cohort (*n* = 66 375)	*P* value	Evusheld cohort (*n* = 391)		
Age (mean ± SD)	58.6 ± 15.4	48.9 ± 18.5	<.0001	58.6 ± 15.5	58.6 ± 18.3	.98
Female gender	195 (47.7%)	36 447 (54.9%)	<.0001	191 (48.8%)	190 (48.5%)	.94
Race						
Hispanic or Latino	15 (3.6%)	2562 (3.8%)	.84	14 (3.5%)	10 (2.5%)	.40
White	354 (86.7%)	52 045 (78.4%)	<.0001	339 (86.7%)	346 (88.4%)	.44
African American	27 (6.6%)	5440 (8.1%)	.24	25 (6.3%)	24 (6.1%)	.88
**IBD type and subtype**						
UC^a^	220 (53.9%)	29 525 (44.4%)	<.0001	214 (53.7%)	229 (57.5%)	.28
Ulcerative proctitis	20 (4.9%)	5457 (8.2%)	.01	20 (5%)	22 (5.5%)	.75
Ulcerative proctosigmoiditis	21 (5.1%)	4,317 (6.5%)	.26	21 (5.2%)	25 (6.2%)	.54
Left-sided colitis	22 (5.3%)	3957 (5.9%)	.62	21 (5.2%)	25 (6.2%)	.54
UC pancolitis	76 (18.6%)	14 045 (21.1%)	.21	76 (19%)	75 (18.8%)	.92
CD^a^	189 (46.3%)	35 376 (53.2%)	.004	187 (46.9%)	174 (43.7%)	.35
CD of small intestine	89 (21.8%)	16 175 (24.3%)	.23	88 (22.1%)	85 (21.3%)	.79
CD of large intestine	104 (25.4%)	18 232 (27.4%)	.37	103 (25.8%)	99 (24.8%)	.74
CD of small and large intestine	89 (21.8%)	17 771 (26.7%)	.02	88 (22.1%)	86 (21.6%)	.86
Hx of COVID-19	89 (21.8%)	3026 (4.5%)	<.0001	82 (20.9%)	85 (21.7%)	.79
**Comorbid diseases**						
Hypertension	231 (56.6%)	15 052 (22.6%)	<.0001	216 (55.2%)	227 (58%)	.42
Hyperlipidemia	175 (42.8%)	12 314 (18.5%)	<.0001	165 (42.1%)	166 (42.4%)	.94
Diabetes mellitus	97 (23.7%)	5630 (8.4%)	<.0001	93 (23.7%)	94 (24%)	.93
Obesity	59 (14.4%)	8576 (12.9%)	.35	57 (14.5%)	55 (14%)	.83
Nicotine dependence	18 (4.4%)	3426 (5.1%)	.49	17 (4.3%)	19 (4.8%)	.73
Ischemic heart disease	69 (16.9%)	3758 (5.6%)	<.0001	64 (16.3%)	72 (18.4%)	.45
Chronic lower respiratory disease	98 (24%)	7148 (10.7%)	<.0001	93 (23.7%)	98 (25%)	.67
Chronic kidney disease	115 (28%)	3245 (4.8%)	<.0001	106 (27.1%)	109 (27.8%)	.81
Heart failure	42 (10.2%)	1804 (2.7%)	<.0001	40 (10.2%)	45 (11.5%)	.56
Cerebrovascular accident	11 (2.6%)	627 (0.94%)	.0003	11 (2.8%)	10 (5.1%)	.82
Rheumatoid arthritis	36 (8.8%)	1514 (2.2%)	<.0001	34 (8.6%)	36 (9.2%)	.80
Ankylosing spondylitis	10 (2.4%)	560 (0.84%)	.0004	10 (2.5%)	10 (2.5%)	1
Psoriasis	14 (3.4%)	1588 (2.3%)	.17	13 (3.3%)	16 (4%)	.57
Systemic lupus erythematous	15 (3.6%)	432 (0.6%)	<.0001	14 (3.5%)	12 (3%)	.69
Human immunodeficiency virus	10 (2.4%)	160 (0.2%)	<.0001	10 (2.5%)	10 (2.5%)	1
Lymphoma/leukemia	96 (23.5%)	596 (0.89%)	<.0001	83 (21.2%)	78 (19.9%)	.65
GI malignancy	17 (4.1%)	731 (1.1%)	<.0001	14 (3.5%)	13 (3.3%)	.84
Skin cancer	28 (6.8%)	1,173 (1.7%)	<.0001	28 (7.1%)	30 (7.6%)	.78
Kidney transplant	11 (2.6%)	13 (0.02%)	<.0001	10 (2.5%)	10 (2.5%)	1
Liver transplant	11 (2.6%)	30 (0.04%)	<.0001	10 (2.5%)	10 (2.5%)	1
Stem cell transplant	35 (8.5%)	61 (0.09%)	<.0001	24 (6.1%)	21 (5.3%)	.64
**Medications** ^b^						
Prednisone	179 (43.8%)	12 329 (18.5%)	<.0001	168 (42.9%)	180 (46%)	.38
Budesonide	44 (10.7%)	6310 (9.5%)	0.38	39 (9.9%)	47 (12%)	.36
Methylprednisolone	152 (37.2%)	8583 (12.9%)	<.0001	140 (35.8%)	163 (41.6%)	.09
Infliximab	25 (6.1%)	7183 (10.8%)	.002	25 (6.3%)	33 (8.4%)	.27
Adalimumab	19 (4.6%)	5907 (8.8%)	.002	19 (4.8%)	21 (5.3%)	.74
Vedolizumab	22 (5.3%)	4532 (6.8%)	.25	21 (5.3%)	22 (5.6%)	.87
Ustekinumab	20 (4.9%)	5529 (8.3%)	.01	20 (5.1%)	21 (5.3%)	.74
Tofacitinib	10 (2.4%)	630 (0.9%)	.001	10 (2.5%)	10 (2.5%)	1
Azathioprine	20 (4.9%)	3890 (5.8%)	.41	19 (4.8%)	21 (5.3%)	.74
Methotrexate	21 (5.1%)	2056 (3%)	.01	19 (4.8%)	20 (5.1%)	.86

Abbreviations: CD, Crohn’s disease; IBD, inflammatory bowel disease; UC, ulcerative colitis. To maintain patient confidentiality, when numbers of events are >1 but <10, TriNetX rounds up events to 10.

^a^Total UC and CD is not 100% due to possible overlap of ICD-10-CM codes in patients.

^b^Medications prescribed within 1 year prior to Evusheld administration.

### Risk of COVID-19

Fifty-five patients (13.4%) in the Evusheld cohort developed COVID-19 within 6 months. After PSM, there was no difference in the risk (aOR 1.35, 95% CI, 0.87–2.10) of COVID-19 in the Evusheld cohort compared to IBD control cohort. Ten (22.7%) patients required hospitalization in the Evusheld cohort. After PSM, there was no difference in the risk (aOR 0.88, 95% CI, 0.33–2.35) of hospitalization between the Evusheld and IBD control cohort. No patients required ICU care or intubation/respiratory support and were deceased in the Evusheld cohort.

There were 9952 patients in the non-IBD control cohort that received Evusheld (mean age 62.3 ± 14.4 years old, female 43.1%). Within 6 months, 592 (5.9%) patients developed COVID-19. After PSM, there was no difference in the risk of COVID-19 (aOR 1.02, 95% CI, 0.67–1.54) between patients with IBD in the Evusheld cohort and non-IBD control cohort that received Evusheld (Table 2).

**Table 2.** Risk of COVID-19 in the IBD Evusheld cohort compared to IBD control cohort and non-IBD control before and after propensity score matching.

Cohort	*N* (%)	Before propensity score matching	*N* (%)	After propensity score matching
		aOR (95% CI)		aOR (95% CI)
IBD Evusheld	55 (13.4%)	2.57 (1.93-3.42)	51 (13%)	1.35 (0.87–2.10)
IBD Control	3793 (5.7%)		39 (10%)	
IBD Evusheld	55 (13.4%)	1.36 (1.01-1.82)	52 (12.9%)	1.02 (0.67–1.54)
Non-IBD control	1021 (10.2%)		51 (12.7%)	

Abbreviations: aOR, adjusted odds ratio; CI, confidence interval; IBD, inflammatory bowel disease.

## Discussion

Our study utilizing a large, US-based multi-institutional prospectively maintained database shows that PrEP with tixagevimab–cilgavimab does not decrease the risk of COVID-19 in patients with IBD compared to patients with IBD who did not receive tixagevimab–cilgavimab after matching for confounding variables. There are 2 possible explanations for this. First, the initial trial of tixagevimab–cilgavimab was conducted when the alpha variant was dominant and was then approved when the delta variant of SARS-CoV-2 was in circulation. Tixagevimab–cilgavimab was approved during the Omicron wave, and it has been shown to be ineffective against the BA.1 and BA.4/5 subvariants.^8^ Second, the FDA revised the initial dosing since the lower dose of 150 mg of tixagevimab and 150 mg of cilgavimab were less active against certain Omicron subvariants. There were 25% of patients in our cohort had received tixagevimab–cilgavimab before this revision. It is unclear how many patients in our cohort received additional doses of tixagevimab–cilgavimab or how many received the now recommended 300 mg of tixagevimab and 300 mg of cilgavimab. The rate of hospitalization of 34.4% is likely an overestimate as TriNetX obfuscates patient counts between ≥1 and <11 and rounds the count to 10 in an attempt to maintain patient anonymity.

There are no prior published studies that have assessed the efficacy of tixagevimab–cilgavimab in patients with IBD. A retrospective cohort study in 43 rheumatologic patients on rituximab who received Evusheld demonstrated a low rate (2.3%) of symptomatic SARS-CoV-2 infection^.9^ Another retrospective cohort study in 21 patients with Rituximab-treated antineutrophilic cytoplasmic antibody vasculitis showed a higher rate (15%) of breakthrough COVID-19.^10^ Both studies had small sample sizes and lacked a comparator group. A retrospective cohort study in solid organ transplant recipient’s demonstrated lower risk of breakthrough SARS-CoV-2 infection in patients who received tixagevimab–cilgavimab compared to patients who did not receive tixagevimab–cilgavimab.^11^ This cohort only included vaccinated patients. The interaction between COVID-19 vaccine and tixagevimab–cilgavimab use in patients with IBD on immunosuppressive therapy remains unclear.

In a previous study by our group, we demonstrated that Paxlovid (nirmatrelvir and ritonavir) use in patients with IBD, and COVID-19 was associated with a decreased risk of hospitalization (aOR 0.35, 95% CI, 0.17–0.74) compared to the IBD control cohort.^12^ In this study, no patients who received nirmatrelvir and ritonavir died while as many as 1.8% of patients who did not receive this treatment died.

Our study has several notable strengths. To our best knowledge, this is the first study that has assessed the efficacy of Evusheld in the IBD population. We utilized a large population-based prospectively maintained database which allows for generalizability of results. We were able to perform PSM between the cohorts to reduce the impact of confounding variables despite the retrospective nature of the database. Our study also has several limitations that warrant consideration when interpreting its findings. We were unable to assess the impact of IBD phenotypes and immunosuppressive therapies, specifically biologic, small molecule, and recent steroid use on risk of COVID-19 in patients with IBD who received tixagevimab–cilgavimab due to small sample sizes. While we attempted to exclude patients who had received COVID-19 vaccine during the follow-up period, there is a possibility that patients could have received vaccine doses outside of HCOs like pharmacies, health centers, travel clinics, or government distribution centers, which could not be captured by the database. Although PSM was performed for all analysis, there still exists the possibility of residual bias due to an unknown confounder. Finally, as with all coding-based studies, results are susceptible to errors in coding, misdiagnosis, or documentation.

In conclusion, our study did not show that Evusheld decreases the risk of COVID-19 in patients with IBD. Our study was performed during the period where Evusheld was approved for earlier variants of COVID-19; however, it is no longer authorized by the FDA.^13^ Prevention of moderate–severe COVID-19 in these patients should focus on vaccination strategies and early COVID-19 therapies. Early interrogation of datasets such as TriNetX may provide real-time data on efficacy of medications approved under emergency-use authorizations and inform clinical decisions.

## Supplementary Material

otad047_suppl_Supplementary_Table

## Data Availability

Data that support the findings of this study are extracted from the TriNetX database and are included within the article.
