# Supplementary material for: Tixagevimab and Cilgavimab (Evusheld) as Pre-exposure Prophylaxis for COVID-19 in Patients With Inflammatory Bowel Disease: A Propensity Matched Cohort Study
Source: Crohns Colitis 360. 2023 Sep 6;5(3):otad047. doi: 10.1093/crocol/otad047 (PMC10482141; doi:10.1093/crocol/otad047)
Supplement: otad047_suppl_Supplementary_Table [file otad047_suppl_supplementary_table.docx]

**Supplementary Table 1: Rxnorm codes of IBD-related medications and CPT codes for administration of Evusheld.**

|  |  |
| --- | --- |
| **IBD medications** | **Rxnorm codes** |
| Prednisone | 8640 |
| Budesonide | 19831 |
| Mesalamine | 52582 |
| Sulfasalazine | 9524 |
| Balsalazide | 18747 |
| Olsalazine | 32385 |
| Azathioprine | 1256 |
| Mercaptopurine | 103 |
| Methotrexate | 6851 |
| Infliximab | 191831 |
| Adalimumab | 327361 |
| Certolizumab | 709271 |
| Golimumab | 819300 |
| Vedolizumab | 1538097 |
| Ustekinumab | 847083 |
| Tofacitinib | 1357536 |
|  |  |
| **Evusheld** | **CPT codes** |
|  |  |
| Injection, tixagevimab and cilgavimab, for the pre-exposure prophylaxis only, for certain adults and pediatric individuals (12 years of age and older weighing at least 40 kg) with no known sars-cov-2 exposure, who either have moderate to severely compromised immune systems or for whom vaccination with any available covid-19 vaccine is not recommended due to a history of severe adverse reaction to a covid-19 vaccine(s) and/or covid-19 vaccine component(s), includes injection and post administration monitoring | M0220 |
| Injection, tixagevimab and cilgavimab, for the pre-exposure prophylaxis only, for certain adults and pediatric individuals (12 years of age and older weighing at least 40 kg) with no known sars-cov-2 exposure, who either have moderate to severely compromised immune systems or for whom vaccination with any available covid-19 vaccine is not recommended due to a history of severe adverse reaction to a covid-19 vaccine(s) and/or covid-19 vaccine component(s), 300 mg | Q0220 |
| Injection, tixagevimab and cilgavimab, for the pre-exposure prophylaxis only, for certain adults and pediatric individuals (12 years of age and older weighing at least 40 kg) with no known sars-cov-2 exposure, who either have moderate to severely compromised immune systems or for whom vaccination with any available covid-19 vaccine is not recommended due to a history of severe adverse reaction to a covid-19 vaccine(s) and/or covid-19 vaccine component(s), includes injection and post administration monitoring in the home or residence; this includes a beneficiary’s home that has been made provider-based to the hospital during covid-19 public health emergency | M0221 |
